# Joint Ancestry and Association Testing in Admixed Individuals

**DOI:** 10.1371/journal.pcbi.1002325

**Published:** 2011-12-22

**Authors:** Daniel Shriner, Adebowale Adeyemo, Charles N. Rotimi

**Affiliations:** Center for Research on Genomics and Global Health, National Human Genome Research Institute, Bethesda, Maryland, United States of America; Columbia University, United States of America

## Abstract

For samples of admixed individuals, it is possible to test for both ancestry effects via admixture mapping and genotype effects via association mapping. Here, we describe a joint test called BMIX that combines admixture and association statistics at single markers. We first perform high-density admixture mapping using local ancestry. We then perform association mapping using stratified regression, wherein for each marker genotypes are stratified by local ancestry. In both stages, we use generalized linear models, providing the advantage that the joint test can be used with any phenotype distribution with an appropriate link function. To define the alternative densities for admixture mapping and association mapping, we describe a method based on autocorrelation to empirically estimate the testing burdens of admixture mapping and association mapping. We then describe a joint test that uses the posterior probabilities from admixture mapping as prior probabilities for association mapping, capitalizing on the reduced testing burden of admixture mapping relative to association mapping. By simulation, we show that BMIX is potentially orders-of-magnitude more powerful than the MIX score, which is currently the most powerful frequentist joint test. We illustrate the gain in power through analysis of fasting plasma glucose among 922 unrelated, non-diabetic, admixed African Americans from the Howard University Family Study. We detected loci at 1q24 and 6q26 as genome-wide significant via admixture mapping; both loci have been independently reported from linkage analysis. Using the association data, we resolved the 1q24 signal into two regions. One region, upstream of the gene *FAM78B*, contains three binding sites for the transcription factor PPARG and two binding sites for HNF1A, both previously implicated in the pathology of type 2 diabetes. The fact that both loci showed ancestry effects may provide novel insight into the genetic architecture of fasting plasma glucose in individuals of African ancestry.

## Author Summary

Most genome-wide association studies performed to date have focused on individuals with European ancestry. Admixed African Americans tend to have disproportionately higher risk for many common, complex diseases. Disease or trait mapping in admixed individuals can benefit from joint analysis of ancestry and genotype effects. We developed a joint test that is more powerful than either admixture mapping of ancestry effects or association mapping of genotype effects performed separately. Our joint test fully capitalizes on the reduced testing burden of admixture mapping relative to association mapping. The test is based on generalized linear models and can be performed using standard statistical software. We illustrate the increased power of the joint test by detecting two loci for fasting plasma glucose in a sample of unrelated African American individuals, neither of which loci was detected as significant by traditional association analysis.

## Introduction

Genome-wide association studies are conventionally performed with an implicit assumption that the prior probability of association is uniform across loci [1]. This assumption can be useful in discovery or hypothesis-generating analysis because the entire genome is scanned rather than limiting the scan to regions selected according to preconceptions of where disease susceptibility loci or trait loci ought to be. However, for admixed samples, this assumption means that any prior evidence from admixture mapping of ancestry effects is completely ignored. Thus, the main motivation of this study is to develop an approach that integrates heterogeneous data types that operate at different scales, *i.e.*, ancestry and genotype effects, in order to maximize statistical power in mapping disease susceptibility loci or trait loci in admixed samples.

Three approaches to combine admixture mapping and association mapping have been described. Tang *et al.* [2] derived a joint test for case-control data under a family-based design based on the transmission-disequilibrium test. Lettre *et al.* [3] described a combined test for samples of unrelated individuals. They performed association mapping by linear regression, modeling local ancestry as an additive covariate [3]. They estimated separate 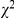 summary statistics for association and local ancestry effects, summed the two statistics, and converted the sum into a combined *p*-value, assuming that the sum was 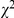-distributed with two degrees of freedom [3]. Two limitations of this approach are that local ancestry and genotype are not independent and the test costs a second degree of freedom. Pasaniuc *et al.* [4] described a combined test that does not suffer from these two limitations. Notably, none of the three tests takes advantage of the reduced testing burden of admixture mapping relative to association mapping. Here, we describe a joint test called BMIX for admixture mapping and association mapping in unrelated individuals that addresses all three issues.

We illustrate application of the joint test by analyzing fasting plasma glucose among 922 non-diabetic, admixed African Americans from the Howard University Family Study (HUFS) conducted in the Washington, D.C metropolitan area. The prevalence of type 2 diabetes (diagnosed mainly on the basis of elevated fasting plasma glucose levels) among adults in the USA is currently 11.3%, ranging from 10.2% among European Americans to 18.7% among African Americans [5]. It is unknown how much genetics contribute to this difference in prevalence. If genetics does contribute, then admixture mapping is an appropriate and efficient approach to use to identify relevant loci [6] and association mapping can be used for fine-mapping.

## Results

### Characterization of Local Ancestry

We first describe the characterization of local ancestry for the 922 admixed African Americans using 797,831 autosomal SNPs. The mean proportion of African ancestry was 0.797 (95% confidence interval 0.770 to 0.819, Supplementary Figure S1). The mean number of ancestry switches per person was 186.0, leading to an estimated 8.1 generations since admixture began [7].

### The Testing Burdens of Admixture Mapping and Association Mapping

**Figure 1:**
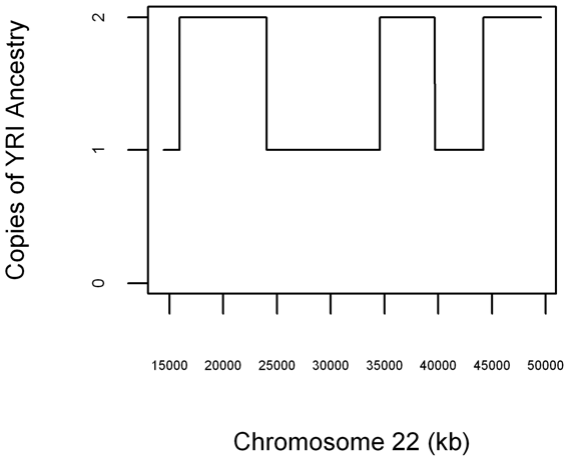
Local ancestry for an admixed African American estimated using LAMPANC version 2.3 [**28**]. For this individual, the chromosome is a mosaic of six segments, reflecting five ancestry switches.

To empirically estimate the testing burdens of admixture mapping and association mapping, we fit autoregressive models and estimated the effective number of tests based on autocorrelation. For example, for the first individual in our sample, there were five ancestry switches along chromosome 22 (Figure 1) and the effective number of tests was 5.5, based on fitting an AR(1) model (see **The Bayesian Model** subsection of **Materials and Methods** for the definition of this model). Summed across autosomes for each individual and averaged across individuals, the effective number of tests for admixture mapping was 368.8. Thus, the genome-wide significance level for admixture mapping was 
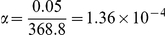
 and the noncentrality parameter for the alternative density for admixture mapping was 21.7. Similarly, the average, genome-wide effective number of tests for association mapping was 345,450.3. Thus, the genome-wide significance level for association mapping was 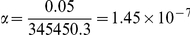 and the noncentrality parameter for the alternative density for association mapping was 37.2. We stress that both testing burden estimates are sample-based (*i.e.*, based only on observed markers rather than all possible markers) and account for correlation for all markers chromosome-wide.

### The Necessity of Controlling for both Local Ancestry and Global Ancestry

Adjusting for global ancestry will not completely control confounding due to local ancestry in association mapping [8], [9]. Wang *et al.* [10] concluded that adjusting for local ancestry is sufficient to control confounding due to either local or global ancestry. However, their conclusion was based on conflating two definitions of local ancestry. The conventional definition of local ancestry is the number of copies of chromosomes inherited from a parental population at a given marker. In the Appendix, Wang *et al.* [10] unconventionally defined local ancestry as either “local ancestry at one locus (referred to as stratification due to local ancestry difference) or the combinations and possibly interactions of ancestries at multiple loci (referred to as stratification due to global ancestry difference)”. An indicator of ancestry defined in the latter way is not equivalent to an indicator of ancestry defined solely by local ancestry. By simulation, we show that adjusting for global ancestry controls confounding due to global ancestry whereas adjusting for local ancestry is insufficient to control confounding due to global ancestry, evident by an inflated type I error rate for association (Supplementary Table S1). Thus, adjusting for local ancestry is necessary to control confounding due to local ancestry and adjusting for global ancestry is necessary to control confounding due to global ancestry.

### Power Analysis

**Figure 2:**
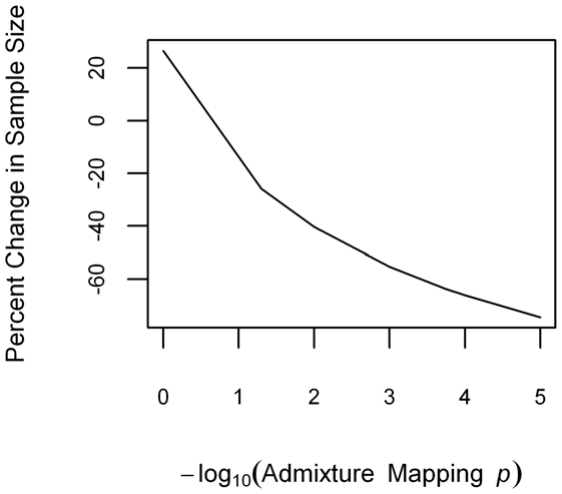
Potential gain in power in association testing using prior admixture mapping evidence. The change in association sample size as a function of *p*-values from admixture mapping was calculated relative to the 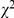 statistic corresponding to genome-wide significance under the uniform prior for association, given that the posterior probability of admixture mapping equals the prior probability of association.

If the posterior probability of a local ancestry effect is smaller than the prior probability of association in the absence of performing admixture mapping, *i.e.*, 
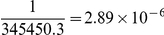
, then more compelling evidence of association is needed to achieve genome-wide significance by our joint test. Conversely, if the posterior probability of a local ancestry effect exceeds 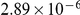, then less compelling evidence of association is needed to achieve genome-wide significance by our joint test. To quantify such behavior, we calculated the change in sample size corresponding to different *p*-values from admixture mapping while maintaining power and the genome-wide significance level for association. As expected, a large *p*-value from admixture mapping implies that the locus is less likely to affect the phenotype, thereby increasing the sample size necessary for association to reach genome-wide significance (Figure 2). The complete absence of local ancestry effects costs the equivalent of a 26.5% increase in the association sample size. Conversely, a small *p*-value from admixture mapping implies that the locus is more likely to affect the phenotype, thereby decreasing the sample size necessary for association to reach genome-wide significance (Figure 2). The break-even point occurs at admixture mapping *p*-values of 0.31, *i.e.*, all admixture mapping *p*-values<0.31 increase the power of subsequent association mapping in our joint test. This break-even point is larger than the point-wise significance level of 0.05, indicating that weak ancestry effects or weakly differentiated markers are capable of improving the power of association mapping. A genome-wide significant *p*-value from admixture mapping equates to a 63.7% reduction in association sample size. For our data, the average prior probability for association mapping conditional on local ancestry was 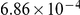, more than two orders of magnitude larger than the prior probability for association mapping in the absence of performing admixture mapping, indicating a substantial gain in average power.

We also compared the average power of our joint test to the MIX score [4]. The MIX score is based on the ancestry odds ratio defined as 
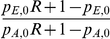
, in which 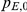 and 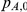 are the allele frequencies among controls in the two parental populations and 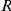 is the allelic odds ratio [4]. We simulated 10,000 independent data sets consisting of one marker for 1,500 controls and 1,500 cases, assigning biologically realistic local ancestry and genotype effect sizes and marginalizing over local ancestry and allele frequencies. To mimic the size of chromosome 22, we set the testing burden of admixture mapping to be 8.067 and the testing burden of association mapping to be 6,039, as estimated from our real data. Correspondingly, the significance level for MIX was set at 
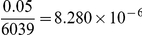
. We first note that the MIX test is valid [4], and that the false positive error rate of our joint test is not different from that of MIX (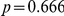, Fisher's exact test, Table 1), indicating that the joint posterior probability of 0.5 is properly calibrated with respect to the admixture mapping and association mapping type I and type II error rates. Our joint test was generally one to two orders of magnitude more powerful than MIX (Table 1). Notably, MIX is less powerful than our joint test when the ancestry and genotype effects oppose each other (*i.e.*, one effect increases risk and the other effect decreases risk). Given that the ratio of the testing burdens for association mapping to admixture mapping for chromosome 22 is smaller than the ratio genome-wide, the gain in power demonstrated by these simulations underestimates the gain in power of BMIX over MIX at the genome-wide scale.

**Table 1:** Average power for our Bayesian joint test compared to the MIX test for simulated case-control data in African Americans.

Local Ancestry Odds Ratio	Genotype Odds Ratio	BMIX	MIX
1.000	1.000	0.0004	0.0002
1.200	1.000	0.0263	0.0004
1.000	1.200	0.0508	0.0289
1.200	1.200	0.1804	0.0670
1.200	0.833	0.1610	0.0220
1.500	1.000	0.7006	0.0070
1.000	1.500	0.2954	0.3588
1.500	1.500	0.8572	0.3777
1.500	0.667	0.8829	0.1850

Data sets consisted of 1,500 cases and 1,500 controls with the average admixture proportion of 80% and population differentiation of 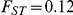 mimicking empirical values for African Americans. Simulations mimicked chromosome 22, such that the significance level was 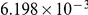 for admixture mapping and 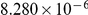 for association mapping.

### High-Density Admixture Mapping for Fasting Plasma Glucose

**Figure 3:**
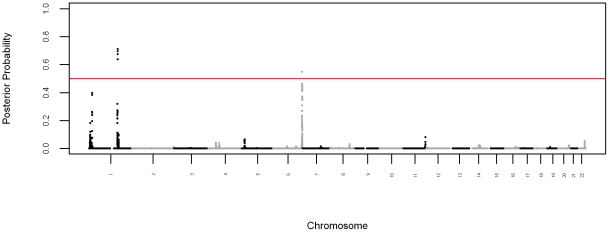
Bayesian Manhattan plot for high-density admixture mapping. The y-axis shows the posterior probability that a locus affects the phenotype. The red line indicates the genome-wide significance level.

We performed admixture mapping for fasting plasma glucose by linearly regressing fasting plasma glucose on local ancestry, adjusted for age, global ancestry, and sex. We detected two genome-wide significant loci (Figure 3), one at chromosome 1q24 (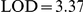) and the other at chromosome 6q26 (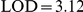). The signal at the 1q24 locus consisted of 93 consecutive genome-wide significant SNPs (posterior probabilities ranging from 0.637 to 0.711) at which increased African ancestry correlated with increased fasting plasma glucose. This locus explained 1.8% of the variance in fasting plasma glucose. The signal at the 6q26 locus consisted of nine consecutive genome-wide significant SNPs at which increased African ancestry correlated with increased fasting plasma glucose. This locus explained 1.7% of the variance in fasting plasma glucose.

### Association Mapping for Fasting Plasma Glucose

**Figure 4:**
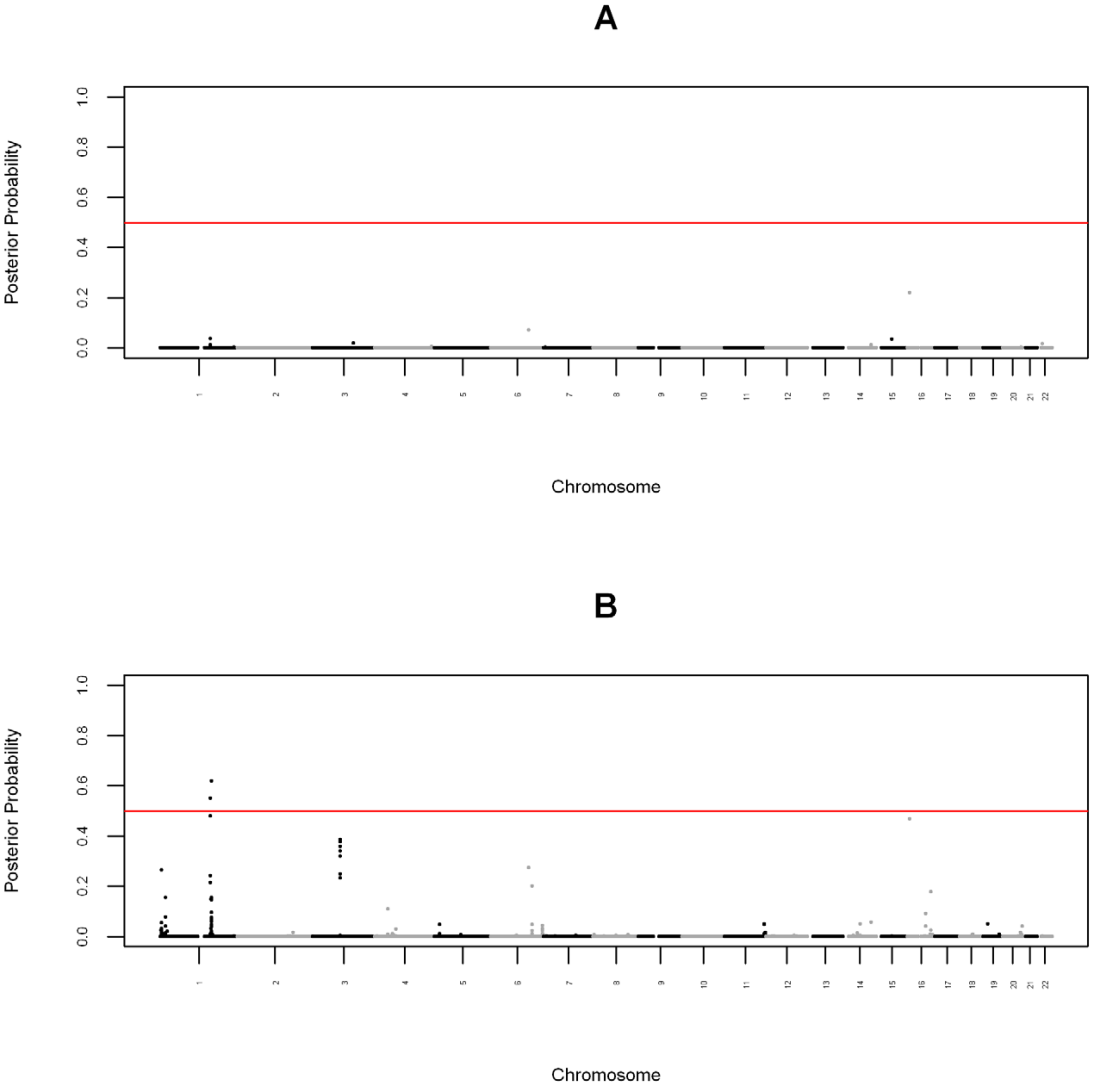
Bayesian Manhattan plot for association. The y-axes indicate the posterior probability that a locus affects the phenotype. The red lines indicate the genome-wide significance level. (A) Association testing under the uniform prior probability. (B) Joint ancestry and association testing.

We performed association mapping for fasting plasma glucose by linearly regressing fasting plasma glucose on genotype stratified by local ancestry, assuming an additive genotype model, adjusted for age, global ancestry, and sex. The genomic control inflation factor was 1.009 (Supplementary Figure S2). We used the posterior probabilities from admixture mapping as the prior probabilities for association mapping. For comparison, using a uniform prior probability of 
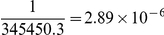
, there were no genome-wide significant findings (Figure 4A). In contrast, using the joint test, we detected two genome-wide significant SNPs, rs7523538 and rs1932355, both at the 1q24 locus detected by admixture mapping (Figure 4B and Supplementary Table S2).

To functionally annotate these two SNPs, we first identified the intervals based on linkage disequilibrium surrounding these two SNPs containing all SNPs with pairwise 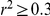. For the top SNP, rs7523538, we identified a 248.6 kb interval from 166,110,586 bp to 166,359,212 bp that lies upstream of the gene *FAM78B*. The FAM78B protein has no known function. However, within the promoter for *FAM78B*, three binding sites for the transcription factor PPARG (from 166,140,317 bp to 166,140,340 bp; from 166,148,656 bp to 166,148,677 bp; and from 166,134,895 bp to 166,134,911 bp) and two binding sites for the transcription factor HNF1A (from 166,153,088 bp to 166,153,103 bp and from 166,153,241 bp to 166,153,256 bp) have been identified (http://www.sabiosciences.com and [11]). Both *PPARG* and *HNF1A* are known susceptibility genes for type 2 diabetes [12]. For the second SNP, rs1932355, we identified a 180.6 kb interval from 163,581,663 bp to 163,762,232 bp. This interval does not overlap any known genes or promoters [11].

## Discussion

We present a joint test of ancestry and association applicable to mapping disease susceptibility loci or trait loci in admixed individuals. Although we proceed through the calculations sequentially by performing admixture mapping first followed by association mapping, equivalence to a joint test can be seen by recognizing that the joint probability of ancestry and association effects equals the product of the probability of an ancestry effect and the probability of association conditional on ancestry. Conditional independence of association given ancestry is necessary for validity of the joint test. For any given marker, admixture mapping is based on the “between” component of local ancestry strata and association mapping is based on the “within” component of local ancestry strata, so that even though both admixture mapping and association mapping are fundamentally based on observed genotypes the data are not used twice. Our joint test is based on generalized linear models and so can be performed with standard statistical software. The admixture mapping step can also accommodate a case-only test [4].

Our joint test of ancestry and association are both genome-wide at equivalent high marker density. Every marker in a sample is tested by both admixture mapping and association mapping, *i.e.*, every marker is tested for genotypic association regardless of the significance of the admixture mapping. Consequently, there is no “winner's curse” [13] in our procedure, because we do not test for association conditional on significance from admixture mapping. As another consequence, our joint test has power to detect loci which do not achieve significance in admixture mapping if the association signal is sufficiently strong. This is in direct contrast to conditional two-stage approaches in which only a subset of markers based on stage one analysis are carried forward to stage two [14], [15]. By design, such conditional approaches have zero power to detect loci that are not selected for analysis in stage two.

Compared to previous approaches, our joint test has several favorable characteristics. The approach of Deo *et al.* [16] is based on sparse panels of ancestry informative markers, whereas high density panels of random markers capture more of the information content regarding ancestry [9]. Lettre *et al.* [3] perform association mapping by linear regression, modeling local ancestry as an additive covariate. However, this approach is not recommended because local ancestry and genotype are correlated. We recommend stratifying genotype by local ancestry because association cannot be confounded by local ancestry within a homogeneous stratum of local ancestry [9]. Perhaps most importantly, our approach fully capitalizes on the reduced testing burden of admixture mapping relative to association mapping while generating a 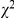 test statistic with only one degree of freedom. For example, using our approach, a *p*-value from admixture mapping of 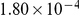 combined with a *p*-value from association mapping of 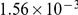 achieves a posterior probability of 0.5. However, using the approach of Lettre *et al.* [3], the posterior probability would be 0.105. The MIX score [4] also fails to capitalize on the reduced testing burden of admixture mapping, resulting in a combined test not as powerful as our joint test. The main limitation of BMIX is that if the local ancestry effect is so strong that the posterior probability after admixture mapping is 1, then the posterior probability will not be updateable with the association data.

By sequentially updating the probability that a locus is a trait locus based on ancestry with the probability that the locus is a trait locus based on genotypic association conditional on ancestry, our procedure estimates the joint probability that a locus has ancestry and association effects. At chromosome 1q24, association mapping resolved the admixture signal into two regions, *i.e.*, association mapping effectively fine-mapped the admixture signal. Chromosome 1q21–q25 is one of the three most often replicated loci from genome-wide linkage analysis for type 2 diabetes, having been replicated in samples of European ancestry (Amish, French, UK, Utah), East Asian ancestry (Chinese, Hong Kong), and Native American ancestry (Pima Indians) [17]. However, candidate gene analyses and dense genotyping have failed to identify common causal variants explaining linkage [17], [18]. Our index SNP rs7523538 is not located in a known functional element but may be in linkage disequilibrium with genetic variation altering transcription factor binding sites, thereby providing a new lead to investigate in terms of locating functional variation as well as determining the functional mechanism. At chromosome 6q26, association mapping eliminated the significance of the admixture signal. One possible interpretation is that the original admixture signal was a false positive finding and the association data appropriately decreased the posterior probability that the 6q26 locus is a trait locus. Alternatively, if the original admixture signal is truly positive, then the association data may be indicating that there is at least one untyped and untagged marker within the interval driving the admixture signal. Given that chromosome 6q26 has been previously linked to insulin sensitivity in a sample of obese African Americans [19], the latter explanation seems more likely.

In summary, we describe a joint test of ancestry and association for mapping disease susceptibility loci and trait loci in admixed individuals. Key properties of our test are that it maintains conditional independence of genotype and local ancestry and that it fully capitalizes on the reduced testing burden of admixture mapping relative to association mapping, making it more powerful than all existing joint tests. Upon application to fasting plasma glucose in African Americans, we identified two loci at genome-wide significance levels, whereas conventional association mapping yielded no new discoveries. Both loci have been identified previously by genome-wide linkage analysis, providing evidence of replication and indicating that linkage analysis, admixture mapping, and association mapping are all converging on the same loci. By taking advantage of fine-mapping afforded by association mapping and background linkage disequilibrium, we resolved one locus into two separate intervals. One of these intervals contains a promoter with multiple binding sites for transcription factors previously implicated in type 2 diabetes. The fact that both loci were discovered via admixture mapping directly implies that the genetic architecture of fasting plasma glucose is different in individuals of European ancestry *vs.* individuals of African ancestry.

## Materials and Methods

### The Bayesian Model

First, we briefly review Bayes' Theorem [20]. Let 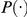 represent a probability and let 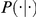 represent a conditional probability. For a given locus, let 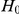 be the hypothesis that the locus does not affect the phenotype and let 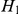 be the hypothesis that the locus does affect the phenotype, subject to the constraint that 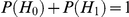. According to Bayes' Theorem, conditional on data 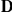, the posterior probability that the locus affects the phenotype is 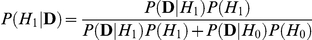. The quantity 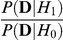 is the marginal likelihood ratio, also known as the Bayes factor, and indicates the strength of evidence for either hypothesis.

Let the likelihood function 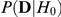 be the 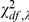 distribution with degrees of freedom 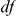 and noncentrality parameter 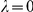 and let the likelihood function 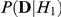 be the 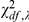 distribution with degrees of freedom 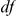 and noncentrality parameter 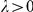. Thus, we can analyze 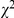 statistics or *p*-values that can be transformed using quantile functions. Given a type I error rate 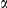 and a type II error rate 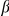, for a one-tailed test, 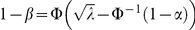 and for a two-tailed test, 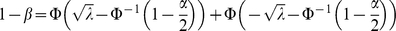, in which 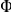 is the standard normal cumulative distribution function and 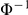 is the standard normal quantile function [21]. As is conventional, we specify power to be 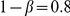. To complete the specification of the alternative densities, we need the type I error rates for admixture mapping and association mapping. We assign the type I error rates to be 0.05 divided by the effective number of tests (*i.e.*, both type I error rates are partially Bonferroni-corrected). We therefore need estimates of the effective number of tests for both admixture mapping and association mapping, which we obtain based on autocorrelation. For admixture mapping, we first estimate the effective number of tests for each chromosome for each individual by fitting an autoregressive model to the vector of local ancestries (0, 1, or 2 chromosomes of African ancestry) and evaluating the spectral density at frequency zero [22]. The notation for an autoregressive model of order 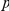 is 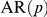 and the model is defined as 
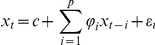
, in which 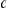 is a constant, 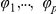 are the parameters, and 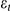 is white noise. The order of the fitted autoregressive model is chosen by minimizing the Akaike information criterion [22]. We sum the effective number of tests for the chromosomes for each individual and then average across individuals. For association mapping, we use the vector of genotypes (recoded as 0, 1, or 2 copies of the minor allele) instead of the local ancestries.

### Bayesian Inference

Two main quantities in Bayesian inference are Bayes factors and posterior probabilities. One advantage of Bayes factors over *p*-values is that the latter accounts only for the density under the null hypothesis whereas the former also accounts for the density under the alternative hypothesis. On the other hand, a disadvantage of Bayes factors is that they, like *p*-values, reflect the probability of the data rather than the probability of a hypothesis. In contrast, posterior probabilities directly measure the probability of a hypothesis. A natural, objective threshold of posterior probabilities is 0.5, which is the point at which the hypothesis favored by the posterior odds switches.

### The Algorithm

The algorithm consists of six steps.

1. Using generalized linear regression, perform admixture mapping by regressing phenotype on local ancestry, adjusting for global ancestry (and other covariates as appropriate). For example, let 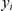 be the observed phenotype for the *i* ^th^ of individual, 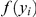 be the link function, 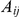 be the local ancestry for the *i* ^th^ individual at the *j* ^th^ marker (*e.g.*, for African Americans, 0, 1, or 2 copies of African chromosomes), and 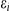 be the residual variance. The basic model for admixture mapping is 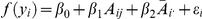, in which 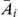 represents the global ancestry for the *i* ^th^ individual (local ancestry averaged across all markers). We require the *p*-value from the test of 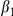.
2. Convert the *p*-values from Step 1 into posterior probabilities. First, transform the *p*-values from admixture mapping into 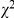 statistics using the quantile function. Then, convert the 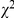 statistics into posterior probabilities using 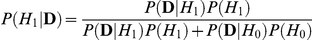, in which 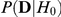 is the density function 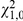, 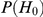 is the prior probability defined by 1 divided by the effective number of tests in admixture mapping, 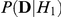 is the density function 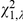 with 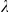 equal to the noncentrality parameter for admixture mapping, and 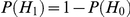.
3. Using generalized linear regression, perform association mapping by regressing phenotype on genotype, stratified by local ancestry, adjusting for global ancestry (and other covariates as appropriate). For example, let 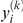 be the observed phenotype for the *i* ^th^ individual in the *k* ^th^ stratum, 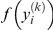 be the link function, 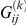 be the genotype for the *i* ^th^ individual in the *k* ^th^ stratum at the *j* ^th^ marker (*e.g.*, 0, 1, or 2 copies of the minor allele), and 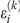 be the residual variance. The basic model for association mapping is 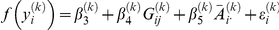. We evaluate each stratum of local ancestry independently, yielding one estimate of 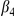 and a standard error per stratum. For African Americans, there are three strata of local ancestry. Stratifying by local ancestry in this step maintains conditional independence of local ancestry and genotype.
4. Combine the regression coefficients for genotype for the strata of local ancestry using inverse variance-weighted fixed effects. The pooled estimate of the genotype effect is given by 
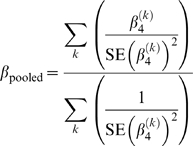
 and the pooled estimate of the standard error is given by 
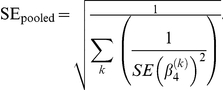

5. Obtain association *p*-values for the pooled estimates of the genotype effects combined over strata. The association test statistic 
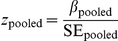
 follows the standard normal distribution.
6. Convert the association *p*-values into posterior probabilities using posterior probabilities from admixture mapping as prior probabilities. First, transform the *p*-values from association mapping into 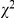 statistics using the quantile function. Then, convert the 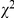 statistics into posterior probabilities using 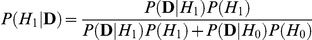, in which 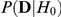 is the density function 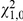, 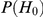 is the prior probability which is equal to the posterior probability from Step 2, 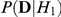 is the density function 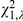 with 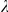 equal to the noncentrality parameter for association mapping, and 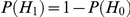.

All calculations were performed in R [23]. Code is provided in Supplementary Text S1.

### Simulating Local Ancestry and Global Ancestry

The procedure to simulate admixed data under a vicariance model has been detailed previously [24], [25]. Briefly, two isolated parental populations were generated with an average value of *F_ST_* of 0.12, mimicking the amount of population differentiation between the African and European ancestors of African Americans. A sample of admixed individuals was generated with an average of 80% of the genome inherited from the first parental population, mimicking the amount of African ancestry in African Americans. For each marker and individual, the genotype was coded as 0, 1, or 2 copies of the derived allele and local ancestry was coded as 0, 1, or 2 copies inherited from the first parental population.

To investigate whether adjusting for local ancestry is sufficient to control confounding due to global ancestry, we simulated two independent SNPs for a sample of 1,000 admixed individuals. The first SNP was the test SNP and the second SNP was untested. We estimated global ancestry by averaging local ancestries.

### Ethics Statement

Ethical approval was obtained from the Howard University Institutional Review Board and written informed consent was obtained from each participant.

### Study Sample

We used BMIX to analyze fasting plasma glucose among 922 non-diabetic, unrelated African Americans from the HUFS (Supplementary Table S3). Fasting plasma glucose was measured from blood samples obtained from participants after an overnight fast using the COBAS INTEGRA Glucose HK Gen.3 test (Roche Diagnostics, Indianapolis, IN). Non-diabetics had fasting plasma glucose levels <126 mg/dL (7.0 mmol/L). Genotyping was performed using the Affymetrix Genome-Wide Human SNP Array 6.0, with quality control as described previously [26], [27]. Local ancestry estimates (0, 1, or 2 chromosomes of African ancestry) were obtained for 797,831 autosomal single nucleotide polymorphisms (SNPs) using LAMPANC version 2.3 [28] and HapMap Phase II+III CEU and YRI reference allele frequencies (http://hapmap.ncbi.nlm.nih.gov/downloads/frequencies/2010-08_phaseIIIII/). We note in passing that we did not include imputation in our study because there is no agreed-upon standard approach to perform imputation in admixed samples at this time. Admixture mapping was performed by linearly regressing fasting plasma glucose on local ancestry, adjusted for age, global ancestry (equal to the individual admixture proportion), and sex. Association mapping was performed assuming an additive genetic model by linearly regressing fasting plasma glucose on genotype stratified by local ancestry, adjusted for age, global ancestry, and sex.

## Supporting Information

Figure S1 Average proportion of African ancestry across the genome, estimated using LAMPANC version 2.3 [28].
(EPS)

Figure S2 Quantile-quantile plot for association *p*-values.
(EPS)

Table S1 Adjusting for local ancestry does not control confounding due to global ancestry.
(DOC)

Table S2 Association results for 1q24 stratified by local ancestry.
(DOC)

Table S3 Clinical characteristics of the 922 participants.
(DOC)

Text S1 R code implementing the BMIX joint test.
(TXT)
