# Supplementary material for: Joint Ancestry and Association Testing in Admixed Individuals
Source: PLoS Comput Biol. 2011 Dec 22;7(12):e1002325. doi: 10.1371/journal.pcbi.1002325 (PMC3245293; doi:10.1371/journal.pcbi.1002325)
Supplement: Table S1 — Adjusting for local ancestry does not control confounding due to global ancestry. (DOC) [file pcbi.1002325.s003.doc]

Supplementary Table S1. Adjusting for local ancestry does not control confounding due to global ancestry.

| Generative Model ^a^ | | Regression Model ^b^ | | |
| --- | --- | --- | --- | --- |
| β_genotype_ | β_global_ | Genotype | Genotype + Global Ancestry | Genotype + Local Ancestry |
| 0 | 0 | 0.055 | 0.054 | 0.054 |
| 1 | 0 | 0.882 | 0.885 | 0.884 |
| 0 | 1 | 0.258 | 0.054 | 0.068 |
| 1 | 1 | 0.841 | 0.881 | 0.874 |
| -1 | 1 | 0.898 | 0.877 | 0.877 |

^a^ The generative model for the phenotype was a linear model with the listed fixed effects for the tested marker, no effects for the untested marker, and noise equal to a random deviate from the standard normal distribution.

^b^ The rejection rates (false positive error rates if $\text{β}_{\text{genotype}}=0$ or power if $\text{β}_{\text{genotype}}\neq0$) for testing genotype association at one marker are shown. The significance level was 0.05.
