# Supplementary material for: Joint Ancestry and Association Testing in Admixed Individuals
Source: PLoS Comput Biol. 2011 Dec 22;7(12):e1002325. doi: 10.1371/journal.pcbi.1002325 (PMC3245293; doi:10.1371/journal.pcbi.1002325)
Supplement: Table S2 — Association results for 1q24 stratified by local ancestry. (DOC) [file pcbi.1002325.s004.doc]

Supplementary Table S2. Association results for 1q24 stratified by local ancestry.

| Characteristic | Local Ancestry ^a^ | rs7523538 | rs1932355 |
| --- | --- | --- | --- |
| Position ^b^ |  | 166,317,814 | 163,714,719 |
| Effect Allele |  | A | G |
| Other Allele |  | G | A |
| Effect Allele Frequency | 0 | 0.900 | 0.708 |
|  | 2 | 0.907 | 0.576 |
| Beta (SE) | 0 | 2.80 (3.97) | 2.42 (3.26) |
|  | 1 | -4.71 (1.73) | 1.12 (0.94) |
|  | 2 | -3.03 (1.05) | 2.88 (0.60) |
|  | Pooled | -3.18 (0.88) | 2.37 (0.50) |
| *P*-value | 0 | 0.485 | 0.463 |
|  | 1 | 6.91×10^-3^ | 0.238 |
|  | 2 | 4.13×10^-3^ | 2.20×10^-6^ |
|  | Pooled | 2.90×10^-4^ | 2.29×10^-6^ |

^a^ “0” indicates two chromosomes of European ancestry, “1” indicates one chromosome of African ancestry and one chromosome of European ancestry, “2” indicates two chromosomes of African ancestry, and “Pooled” indicates the meta-analysis combined over the three strata of local ancestry.

^b^ Positions are based on GRCh37.
