# Supplementary material for: Joint Ancestry and Association Testing in Admixed Individuals
Source: PLoS Comput Biol. 2011 Dec 22;7(12):e1002325. doi: 10.1371/journal.pcbi.1002325 (PMC3245293; doi:10.1371/journal.pcbi.1002325)
Supplement: Table S3 — Clinical characteristics of the 922 participants. (DOC) [file pcbi.1002325.s005.doc]

Supplementary Table S3. Clinical characteristics of the 922 participants.

| Variable | Mean (SD) or Percent |
| --- | --- |
| Age (yr) | 46.5 (13.0) |
| Female | 58.0 |
| Fasting plasma glucose (mg/dL) | 85.8 (11.1) |
